# ssc-miR-451 Regulates Porcine Primary Adipocyte Differentiation by Targeting ACACA

**DOI:** 10.3390/ani10101891

**Published:** 2020-10-16

**Authors:** Mailin Gan, Linyuan Shen, Yuan Fan, Ya Tan, Lin Liu, Lei Chen, Ye Zhao, Lili Niu, Guoqing Tang, Qiang Li, Xu Xu, Tinghuan Zhang, Xuewei Li, Shunhua Zhang, Li Zhu

**Affiliations:** 1 College of Animal Science and Technology, Sichuan Agricultural University, Chengdu 611130, China; ganmailin@stu.sicau.edu.cn (M.G.); shenlinyuan@sicau.edu.cn (L.S.); fanyuan@stu.sicau.edu.cn (Y.F.); tanya@stu.sicau.edu.cn (Y.T.); 2018302012@stu.sicau.edu.cn (L.L.); chenlei815918@sicau.edu.cn (L.C.); zhye@sicau.edu.cn (Y.Z.); niulili@sicau.edu.cn (L.N.); 13541@sicau.edu.cn (G.T.); xuewei.li@sicau.edu.cn (X.L.); 2 Farm Animal Genetic Resources Exploration and Innovation Key Laboratory of Sichuan Province, Sichuan Agricultural University, Chengdu 611130, China; 3 Institute of Animal Husbandry and Veterinary, Guizhou Academy of Agricultural Science, Guiyang 550005, China; 4 Sichuan Province General Station of Animal Husbandry, Chengdu 611130, China; liqiang4071@163.com (Q.L.); xx908517582@163.com (X.X.); 5 Chongqing Academy of Animal Science, Rongchang County, Chongqing 402460, China; S20112715@stu.sicau.edu.cn

**Keywords:** ssc-miR-451, adipocyte differentiation, meat quality, fatty acid, ACACA

## Abstract

**Simple Summary:**

miR-451 has been extensively studied in humans and model animals. However, there are no reports of miR-451 in pigs, and it is unclear whether miR-451 is related to adipose tissue development. In this work, we first reported the effects of ssc-miR-451 on pig adipose development and meat quality. We focused on the expression and functions of miR-451 in porcine primary adipocyte and pork quality. Porcine primary adipocyte transfection test and dual luciferase reporting system suggested that ssc-miR-451 may inhibit lipid deposition by inhibiting Acetyl-CoA carboxylase alpha (ACACA) expression. Correlation analysis negatively correlated miR-451 expression with intramuscular fat content and positively correlated ACACA expression with intramuscular fat content. Further analysis of fatty acid composition revealed that pigs with high expression of ssc-miR-451 had higher monounsaturated fatty acid and lower polyunsaturated fatty acid.

**Abstract:**

miRNA is a small non-coding RNA, which plays an important role in diverse biological processes. In the present study, we explore the effect of ssc-miR-451 on porcine adipose development and meat quality. We observed that ssc-miR-451 was downregulated during porcine primary adipocyte differentiation. Overexpression of ssc-miR-451 inhibited adipogenic differentiation, while inhibition of ssc-miR-451 promoted adipogenic differentiation. The dual luciferase reporter system indicated Acetyl-CoA carboxylase alpha (ACACA) as a target gene of ssc-miR-451. Correlation analysis negatively correlated miR-451 expression with intramuscular fat content (IMF) and positively correlated ACACA expression with IMF. Further analysis of fatty acid composition revealed that pigs with high expression of ssc-miR-451 had higher monounsaturated fatty acid (MUFA) and lower polyunsaturated fatty acid (PUFA). Taken together, our study suggests that ssc-miR-451 regulates lipid deposition and fatty acid composition by targeting ACACA, and ssc-miR-451 may serve as a potential genetic marker to improve pork quality.

## 1. Introduction

People in most countries and regions around the world have a tradition of eating pork. For a long time, global pork production and consumption have exceeded 30% of total meat production and consumption (FAO, http://www.fao.org/faostat/zh/#data/QL). Pork quality is closely related to human health and food safety. Adipose tissue as an important component of pork is not only closely related to pig health, but also meat quality. For pigs, adipose tissue is the main energy storage organ as well as an important metabolic and endocrine organ [1], which affects the growth and health of the pig. For farmers, the fat development of pigs is closely related to the feeding time, feed consumption, and pork quality, which is directly related to the feeding cost and selling price. For consumers, adipose tissue will affect the appearance, flavor, and taste of pork. Understanding the regulation of pig fat tissue development and metabolic regulation is of great significance for pig production and disease treatment [2].

The development and metabolism of adipose tissue is regulated by multiple factors. CCAAT enhancer binding protein alpha (C/EBPα) [3], Acetyl-CoA carboxylase alpha (ACACA), fatty acid synthase (FASN), and stearoyl-CoA desaturase (SCD1) are widely reported to be involved in adipose tissue development and lipid metabolism [4]. These candidate genes related to pig fat content have also been used to study whether they can be used as molecular markers for selection of pork quality traits [5]. In recent years, epigenetic molecules have received widespread attention in gene–environment interaction studies. MicroRNA (miRNA), an important epigenetic molecule, play crucial roles in various biological processes in animals [6]. Studies have shown the role of miR-152, miR-10b, miR-143, and few other miRNAs in adipose tissue development [7].

miR-451 has been extensively studied in humans and model animals. miR-451 participates in the occurrence of cancer by regulating YWHAZ [8], PSMB8 [9], CARF [10], and so on [11]. However, there are no in-depth reports of miR-451 in pigs [12], and it is unclear whether miR-451 is related to adipose tissue development.

The aim of this study was to understand the regulatory role of miR-451 in the process of pig fat development, and whether miR-451 can be used as a molecular marker that affects pork quality indicators. We conducted in vitro experiments using primary cells and analyzed the correlation between the expression of miR-451 and pork quality traits. We found that miR-451 may regulate the differentiation of porcine primary adipocyte through ACACA and affect the fat content and fatty acid composition of pork.

## 2. Materials and Methods

All experiments were conducted in accordance with the requirements and standards of the Sichuan Agricultural University Ethics Committee (Sichuan, China, No. DKY-B20131403).

### 2.1. Animals and Treatments

A total of 30 commercial pigs and 3 piglets were used in this study. All pigs fasted for 24 h and were euthanized with electric shocks and bleeding.

Commercial pigs: (1) 18 DLY (Duroc × Landrace × Yorkshire) pigs including 9 males and 9 females, slaughtered at 180 days of age. After fasting with ad libitum access to water for 24 h, pigs were electrically stunned and exsanguinated. About 10 g of the longissimus dorsi muscle from the last rib were collected, immediately placed in liquid nitrogen, and stored at −80 °C for RNA extraction and fatty acid analysis. (2) 6 YP pigs (Yorkshire) and 6 QYP pigs (Qingyu), these pigs were used in the analysis of fatty acid composition in this study, and the pig samples are the same as those used in our previous article [13].

Piglets: Three 3-week-old male DLY piglets. After bleeding, the piglet’s subcutaneous adipose tissue was separated in a sterile environment for primary cell culture. At the same time, about 1 g of piglet tissue samples from each organ (skin (pig’s buttocks skin), adipose (back subcutaneous fat), muscle (longissimus dorsi muscle at 6 to 7 ribs), heart, lung, kidney, spleen, and liver) were taken and stored at −80 °C for Rt-qPCR.

### 2.2. Porcine Primary Adipocyte Culture and Cell Transfection

Porcine primary adipocytes were isolated from subcutaneous adipose tissue at the back of 3-week-old piglets, and the cells were cultured with DMEM/F12 cell culture medium (Gibco, Grand Island, NY, USA). Cells were subcultured at about 70% confluence. Adipogenic differentiation was performed using F3 generation cells. Cells were maintained in growth medium for two days after attaining 100% confluence and then in differentiation medium I (1 μM Dexamethasone (DEX, Solarbio, Beijing, China), 0.5 mm 3-Isobutyl-1-methylxanthine (IBMX, Sigma, St. Louis, MO, USA), 10 μg/mL Insulin (Sigma) for 14 days, and in differentiation medium II (10 μg/mL Insulin) for 4 days [14]. After differentiation for 14 days, cells were transfected with ssc-miR-451 mimic, inhibitor, or negative control (Ribobio, Guangzhou, China) using Lipofectamine 3000 (Invitrogen, Guangzhou, China). In addition, the primary cells induced to differentiate for 4d (early differentiation) and 20d (late differentiation) were collected to analyze the expression characteristics of miR-451 and adipogenesis-related genes (C/EBPα, FASN, SCD1, ACACA).

### 2.3. RNA Extraction and Quantitative Real-Time PCR

The quantitative real-time PCR (RT-qPCR) operation follows the MIQE guidelines. RNA was extracted from cells using TRIzol (TaKaRa, Dalian, China). To each well of the 12-well culture plate (or 50 mg of tissue samples), 1 mL of TRIzol was added and lyse it for 5 min, and RNA was extracted according to the manufacturer’s instructions. RNA was reverse transcribed into cDNA using microRNA and mRNA reverse transcription kit (TaKaRa). RT-qPCR was performed using the SYBR Premix Ex Taq kit (TaKaRa) [15] in a CFX96 real-time PCR detection system (Bio-Rad) [16], and using the 2^−ΔΔCt^ method. β-actin and U6 genes were used as the internal controls to normalize mRNA and miRNA levels, respectively. The primer sequences used for RT-qPCR are shown in Table 1.

### 2.4. Prediction of the Binding Site of miR-451 and ACACA

Download the mature sequence of ssc-miR-451 from miRBase (http://www.mirbase.org/), enter the sequence information of ssc-miR-451 and the sequence information of ACACA on the work page of RNAhybird (https://bibiserv.cebitec.uni-bielefeld.de/rnahybrid/. The hybridization is performed in a kind of domain mode, i.e., the short sequence is hybridized to the best fitting part of the long one. The tool is primarily meant as a means for microRNA target prediction. The detailed operation process refers to the tutorial on the webpage) and use the miRNA seed sequence (position 2–8) for matching to obtain miR-451 and potential binding site and free energy of ACACA. Then, select a site with low free energy and complementary pairing with the seed sequence base, and use the dual luciferase reporter system for verification.

### 2.5. Luciferase Reporter Assay

The wild-type or mutant CDS (coding sequence) region of ACACA was inserted into psiCHECK™-2 vector (Promega, Madison, WI, USA). These plasmids were sequenced (TsingKe Biotech) to confirm the presence and orientation of the insert. PK15 cells were co-transfected with the miR-451 mimic or control (Ribobio) and constructs harboring the corresponding wt or mut ACACA psiCHECK™-2 vector, and relative luciferase activity was determined [17].

### 2.6. Oil Red O Staining

When the pig primary cells were differentiated for 20 days, they were fixed with 4% paraformaldehyde for 1h, then stained with 5% oil red O (Sigma, St. Louis, MO, USA) for 1 h, washed with PBS, and photographed.

### 2.7. Measurement of Meat Quality

Meat quality traits were determined as described previously [18,19]. Meat color including lightness (L*), redness (a*), and yellowness (b*) were determined at 24 h postmortem on longissimus dorsi muscle using Minolta CR-300 colorimeter (Minolta Camera, Osaka, Japan). Drip loss, cooking loss, and sheer force of longissimus dorsi muscle samples were measured as previously outlined [19].

### 2.8. Chemical Composition

The crude protein content (CP) of longissimus dorsi muscle was determined by Kjeldahl method, and the intramuscular fat content (IMF) of longissimus dorsi muscle was determined by Soxhlet extraction method. Ash content was determined as the residue remaining after incineration of organic materials in a high temperature furnace at 550–600 °C [20].

### 2.9. Analysis of Fatty Acids

Tissue sample (100 mg) was homogenized with 2 mL of n-hexane and shaken at 50 °C for 30 min. To this, 3 mL of KOH methanol solution (0.4 mol/L) was added and shaken for 30 min at 50 °C. Further, 1 mL of water and 2 mL of n-hexane were added and mixed. The mixture was allowed to stand for stratification, the upper layer was collected, and the fatty acid was detected by gas chromatography-mass spectrometry. Column: DB-23 (30 m × 320 um × 0.25 um); carrier gas: helium; inlet temperature 250 °C; detector temperature 230 °C; the oven temperature was programmed as follows: An original temperature of 50 °C for 1 min, increased to 175 °C at 25 °C/min, increased to 230 °C at 4 °C/min, and maintained at this temperature for 24.75 min. The split ratio was 1:5, and the injection volume was 1 μL. (GC-MS 7890B-5977A, Agilent, CA, USA) [13].

### 2.10. Statistical Analysis

Data expressed are mean ± standard deviation (SD). One-way analysis of variance (ANOVA) was used to determine statistically significant differences among the mean values using SPSS software (SPSS 20.0, SPSS Inc., NC, USA). Differences were considered statistically significant at *p* < 0.05.

## 3. Results

### 3.1. Downregulation of ssc-miR-451 during Porcine Primary Adipocyte Differentiation

**Figure 1:**
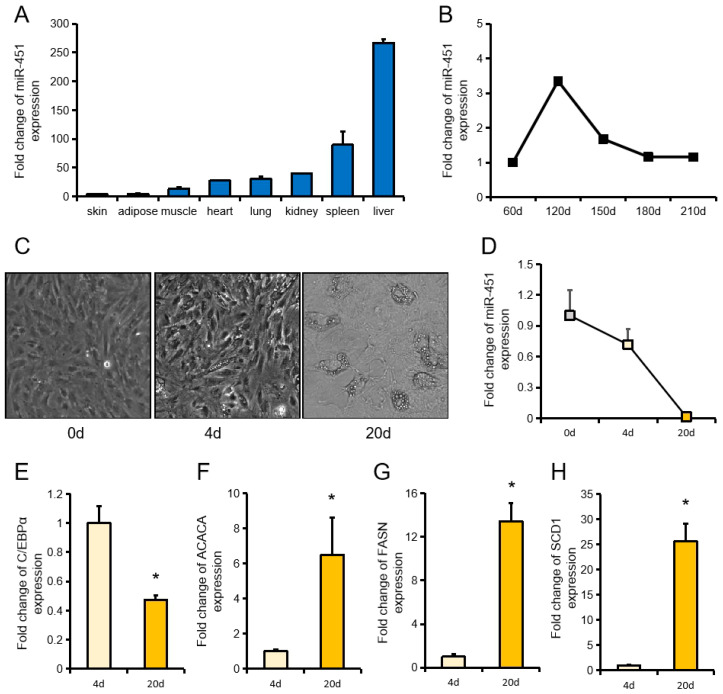
Expression characteristics of ssc-miR-451. (**A**) ssc-miR-451 tissue expression profile on pigs (Three 3-week-old meal Duroc × Landrace × Yorkshire (DLY) piglets), the expression of mir-451 in skin tissue was designated as “1”. (**B**) Expression of miR-451 in large white pigs of different days, data source [21]. (**C**) Diagram of different stages of differentiation of porcine primary adipocytes. (**D**) Changes of ssc-miR-451 expression in porcine primary adipocytes. (**E**–**H**) Expression of lipid deposition-related genes during differentiation of pig primary cells at 4d and 20d, (**E**): CCAAT enhancer binding protein alpha (C/EBPα), (**F**): Acetyl-CoA carboxylase alpha (ACACA), (**G**): Fatty acid synthase (FASN), (**H**): Stearoyl-CoA desaturase (SCD1). All results are presented as means ± SEM. *n =* 3. * *p* < 0.05.

The tissue expression profile of miR-451 on pigs showed that ssc-miR-451 was highest expressed in liver and lowest in skin (Figure 1A). We used the data from previous studies to obtain the expression changes of miR-451 in large white pigs at different days of age (Figure 1B). In vitro experiments revealed gradual downregulation of ssc-miR-451 during porcine primary adipocyte differentiation (Figure 1C,D). Further analysis revealed that the genes related to fat deposition, ACACA, FASN, and SCD1, were significantly higher at the end of differentiation than at the beginning of differentiation, while the expression trend of C/EBPα was reversed (Figure 1E–H).

### 3.2. ssc-miR-451 Inhibits Porcine Pre-Adipocyte Differentiation

**Figure 2:**
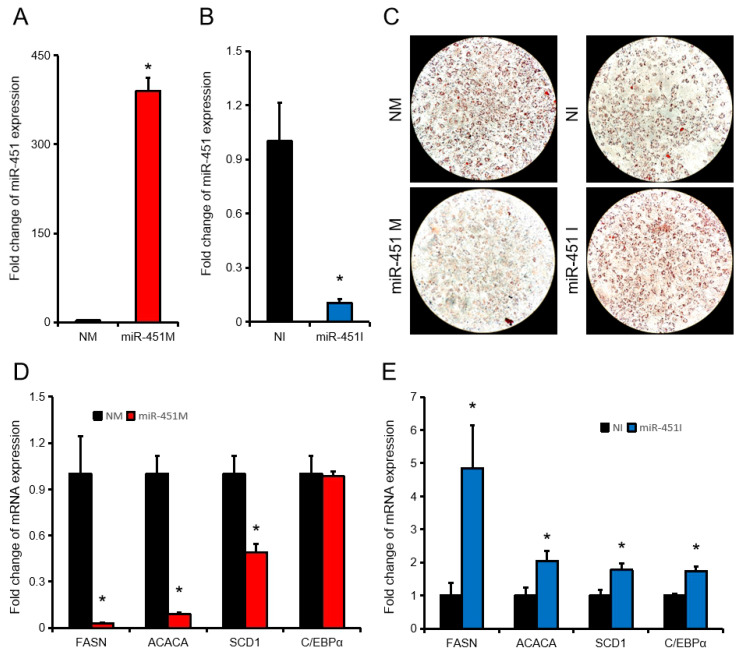
ssc-miR-451 regulates the differentiation of porcine primary adipocytes. (**A**,**B**) miR-451 expression in porcine primary adipocytes after transfection with miR-451 mimic (**A**) and inhibitor (**B**). (**C**) Oil red staining of porcine primary adipocytes. (**D**,**E**) The expression of FASN, ACACA, SCD1, and C/EBPα in porcine primary adipocytes after transfection with miR-451 mimic (**D**) and inhibitor (**E**). All results are presented as means ± SEM. *n =* 3. * *p* < 0.05.

To further investigate the effect of ssc-miR-451 on porcine adipose development, we over-expressed ssc-miR-451 (390 folds) in porcine primary adipocytes (Figure 2A). Transfection of ssc-miR-451 inhibitor (miR-451I) in porcine primary adipocytes successfully inhibited ssc-miR-451 expression by approximately 90% compared with the control group (NI) (Figure 2B). Over-expression of ssc-miR-451 significantly inhibited the formation of lipid droplets in porcine primary adipocytes, while inhibiting miR-451 significantly promoted the differentiation of porcine primary adipocytes (Figure 2C). Compared with the control group, ssc-mir-451 over-expression significantly inhibited the expression of FASN, ACACA, and SCD1 genes, which promoted lipid synthesis. Inhibition of ssc-miR-451 significantly promoted the expression of FASN, ACACA, SCD1, and C/EBPα (Figure 2D–E). These results suggest that ssc-miR-451 can inhibit lipid accumulation in porcine primary cells in vitro.

### 3.3. ssc-miR-451 Tragets ACACA in Porcine

**Figure 3:**
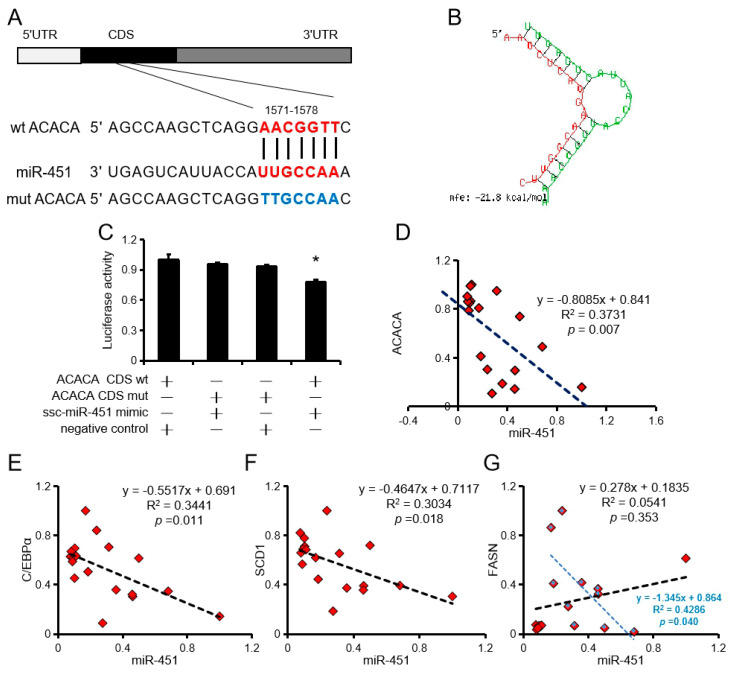
ACACA is a target gene of ssc-miR-451. (**A**,**B**) Binding site (**A**) and free energy (**B**) of ssc-miR-451 and ACACA. (**C**) Pooled ssc-miR-451 mimic, wt-ACACA CDS, or mut-ACACA CDS was co-transfected with the psiCHECK™-2 vector into PK15 cells, and normalized luciferase activity was assayed. *n =* 3. * *p <* 0.05. (**D**) Correlation analysis of porcine primary adipocyte ssc-miR-451 and ACACA, *n =* 18. (**E**) Correlation analysis of porcine primary adipocyte ssc-miR-451 and C/EBPα, *n =* 18. (**F**) Correlation analysis of porcine primary adipocyte ssc-miR-451 and SCD1, *n =* 18. (**G**) Correlation analysis of porcine primary adipocyte ssc-miR-451 and FASN, *n =* 18. The blue mark is the correlation analysis after removing the discrete points, *n =* 10.

RNAhybrid (https://bibiserv.cebitec.uni-bielefeld.de/rnahybrid/) analysis revealed two binding sites for ssc-miR-451 in the CDS region of AACCA (Figure 3A,B). Dual luciferase reporter system analysis revealed that ssc-miR-451 can only bind to the first site of ACACA (Figure 3C). In addition, ssc-miR-451 expression negatively correlated with ACACA, C/EBPα, and SCD1 expression in porcine primary adipocytes but had no significant correlation with FASN (Figure 3D–G).

### 3.4. Correlation of ssc-miR-451 and ACACA with Pork Quality Traits

**Figure 4:**
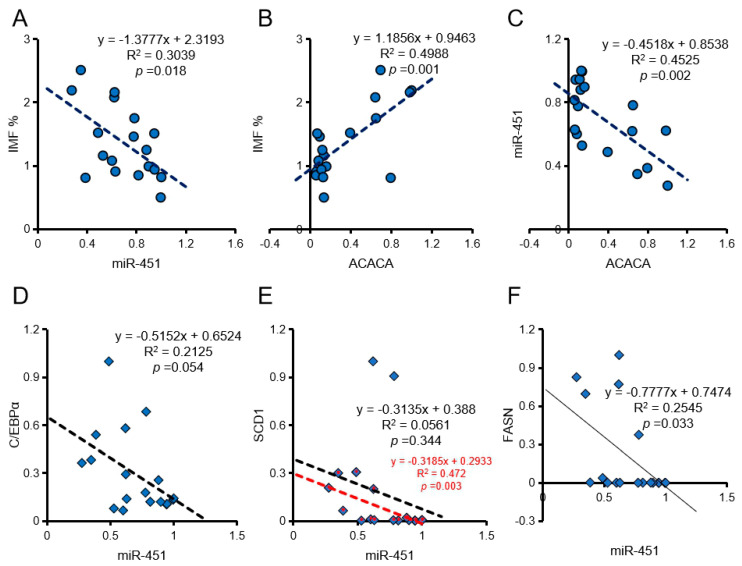
Effect of ssc-miR-451 on intramuscular fat content (IMF) of longissimus dorsi. (**A**,**B**) IMF correlates with miR-451 and ACACA expression in longissimus dorsi, *n =* 18. (**C**) The expression of miR-451 correlates with ACACA in pork, *n =* 18. (**D**) The expression of miR-451 correlates with C/EBPα in pork, *n =* 18. (**E**) The expression of miR-451 correlates withSCD1 in pork, *n =* 18. The red mark is the correlation analysis after removing the discrete points, *n =* 16. (**F**) The expression of miR-451 correlates with FASN in pork, *n =* 18. The data of correlation analysis were from 18 DLY pigs (half barrows and half gilts).

Correlation analysis showed that ssc-miR-451 expression positively correlated with b value and negatively correlated with cooking loss and IMF (Table 2, Figure 4A). The expression of ACACA positively correlated with marbling score and IMF (Table 2, Figure 4B). Interestingly, the expression of ssc-miR-451 and ACACA also showed a significant negative correlation in vivo (Figure 4C). In addition, ssc-miR-451 also has a negative correlation with C/EBPα and FASN in pig longissimus dorsi muscle (Figure 4D–F).

**Figure 5:**
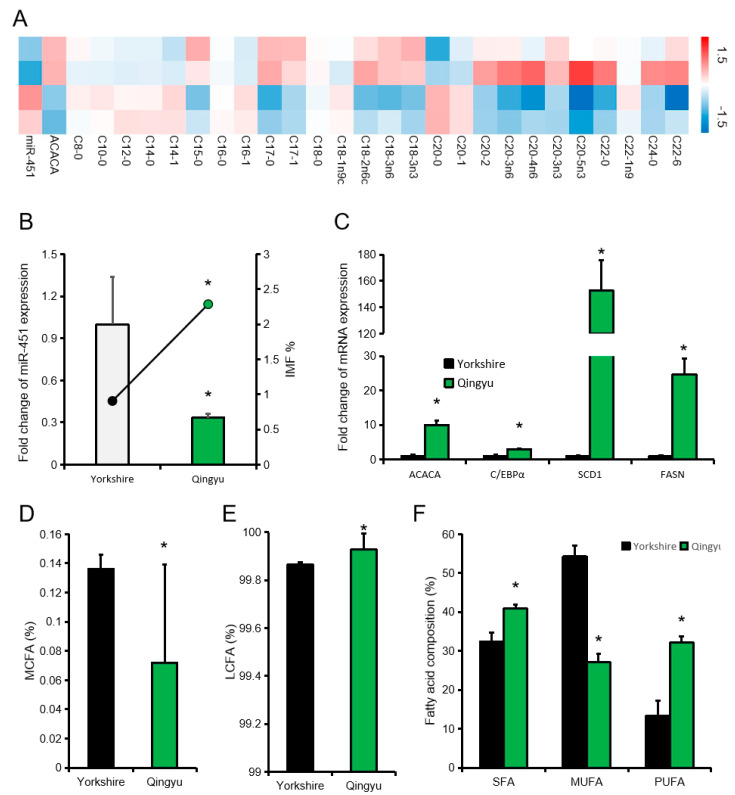
Effect of ssc-miR-451 on fatty acid composition of longissimus dorsi. (**A**) Composition of 26 fatty acids of longissimus dorsi muscle. (**B**) Intramuscular fat content and miR-451 expression levels in Qingyu pigs and Yorkshire pigs, *n =* 3. (**C**) Expression of candidate genes for fat deposition in two pig breeds, *n =* 3. (**D**) Composition of medium chain fatty acids (MCFA) of longissimus dorsi muscle in Qingyu Pigs and Yorkshire Pigs. *n =* 6. (**E**) Composition of long chain fatty acids (LCFA) of longissimus dorsi muscle in Qingyu Pigs and Yorkshire Pigs *n =* 6. (**F**) Fatty acid composition of longissimus dorsi muscle in Qingyu Pigs and Yorkshire Pigs. *n =* 6. * *p* < 0.05. The data is from our previous article [13].

In addition, we compared the fatty acid composition of pigs with low or high expression levels of ssc-miR-451 (high expression group is twice as low expression group). The heat map showed that there was a significant difference in fatty acid composition between the high expression miR-451 group and the low expression miR-451 group. We further selected Qingyu pig (Chinese traditional fat pig breed) and Yorkshire pig to study the effect of miR-451 on fatty acid composition of different pig breeds. The expression level of miR-451 in Qingyu pigs was significantly lower than Yorkshire pigs, while the expression levels of candidate genes related to fat deposition were significantly higher than Yorkshire pigs (Figure 5B,C). Compared with Yorkshire pigs (High miR-451 expression), Qingyu pigs (Low miR-451 expression) also have lower LCFA and higher PUFA (Figure 5D–F).

## 4. Discussion

miRNA is a type of short, single-chain, non-coding RNA. It usually regulates the expression of genes after transcription, and participates in a variety of signal pathways, playing an important biological role [22]. Analysis of the mature sequence of miR-451 revealed that pigs are identical to rats, mice, humans, and zebrafish, but different from cattle. It is worth noting that the miRNA executive function mainly depends on the seed sequence, and the miR-451 seed sequences of the six species are the same, suggesting that they may have similar functions [23]. In humans and other model animals, abnormal expression of miR-451 has been related to the occurrence and development of various fat metabolism-related diseases [24]. Adipose tissue is the major energy storage organ as well as an important endocrine organ, which participates in immune and inflammatory response. ssc-miR-451 is located on pig chromosome 12. The current study on ssc-miR-451 in pigs focuses on sequencing results, and there is still no systematic research on its function.

Lipid deposition in adipocytes is regulated by a series of molecular elements. C/EBPα is expressed in a large amount at the terminal stage of adipocyte differentiation, which can stop the proliferation of cells and show a complete differentiation state [3]. ACACA mainly carboxylates acetyl-CoA to malonyl-CoA, and FASN catalyzes malonyl-CoA to synthesize palmitoyl-CoA, so ACACA and FASN are rate-limiting enzymes for fatty acid synthesis. SCD1 is the rate-limiting enzyme for fatty acid desaturation [4]. Therefore, ACACA, FASN, and SCD1 are often used as markers to evaluate the degree of adipocyte differentiation and candidate genes that affect IMF in animals [25]. Overexpression of miR-451 in pig primary cells, the expression levels of FASN, ACACA, and SCD1 all decreased significantly, while inhibition of miR-451, FASN, ACACA, SCD1, and C/EBPα expression levels increased significantly. The results showed that inhibiting miR-451 may promote adipocyte differentiation and accelerate adipocyte fatty acid synthesis.

The execution of miRNA functions depends on its target genes. This paper reveals ACACA as a target gene of ssc-miR-451. ACACA is associated with long-chain fatty acid synthesis [26] and is used as a potential molecular marker to determine pork quality [27]. In addition, we also found that over-expression or inhibition of miR-451 in pig primary cells, FANS, ACACA, and SCD1 all have significant changes, but over-expression of miR-451 does not affect the expression of C/EBPα, so we speculate that the expression of miR-451 is more related to fatty acid synthesis and fatty acid composition. Analysis of fatty acid composition showed that pigs with higher ssc-miR-451 expression levels had higher MCFA and MUFA contents and lower PUFA content, both within the same pig breed and between different Chinese and Western pig breeds [13]. We speculate that high levels of miR-451 inhibited ACACA expression, which is a rate-limiting enzyme for de novo synthesis of long-chain fatty acids in pig muscles [28]. Therefore, when miR-451 expression levels in muscles are higher, the proportion of LCFA in muscles decreases and MCFA increases. The changes in MUFA and PUFA content may be related to SCD1, a fatty acid desaturase. We detected that pigs with high miR-451 expression levels have lower SCD1 expression levels. Similar to previous reports, we found that pigs with high SCD1 have higher PUFA, but the results of MUFA are different from previous reports. At present, the difference in SCD1 gene expression between breeds has not been extensively studied [29], and the content of MUFA and PUFA is also affected by feed, age, and pig breeds. At the same time, there is a lack of research on the interaction between miR-451 and SCD1. Therefore, the effect of miR-451 on MUFA and PUFA cannot be explained well. FASN and SCD1 have been reported to have single nucleotide polymorphisms (SNP) sites that affect pork quality [25,30,31]. In this study, whether miR-451 or FASN or SCD1 have SNP sites that affect the results of correlation analysis is not known, but it deserves further attention.

Previous studies have found that part of intramuscular fat may migrate from other parts [32], and miR-451 has been reported to regulate cell migration. miR-451 regulates cell migration via macrophage migration inhibitory factor [33] and activating transcription factor 2 [34]. Recently, researchers have observed the migration of the intramuscular fat from the adipose stromal cells from subcutaneous adipose in mice [32]. In addition, many studies have positively correlated backfat thickness with intramuscular fat [35]. Researchers tried to increase IMF while reducing backfat thickness [36]; however, there are still many aspects to be explored, such as the role of miR-451 of pig subcutaneous fat in the formation of intramuscular fat and the relationship between miR-451 and pig adipose development, meat quality, and inflammation. Our present findings provide new insights for further research on improve pork quality.

## 5. Conclusions

In summary, this work focused on the expression and functions of miR-451 in porcine primary adipocyte and pork quality. Our results suggested that ssc-miR-451 may inhibit lipid deposition and regulate IMF and fatty acid composition by inhibiting ACACA expression. Our findings provided new insights and references for the further genetic researches on improving of pork quality.

## Figures and Tables

**Table 1:** The primer sequences used for qRT-PCR (F: Forward, R: Reverse).

Gene	Primer Sequence (5′–3′)	TM/°C	Efficiency
C/EBPα	F-CAAGAACAGCAACGAGTACCG	59	0.94
	R-GTCACTGGTCAACTCCAGCAC		
FASN	F-TCGTGGGCTACAGCATGATA	60.7	0.93
	R-TTAGGCTTCAGCAGGACGTT		
SCD1	F-ACACTTGGGAGCCCTGTATG	60	0.91
	R-GGGCAGTCGAGCTTTGTAAG		
ACACA	F-ACCTCTGGAGTTGAACCAGC	60	0.96
	R-GTGTAAGGCCAAGCCATCCT		
β-actin	F-AAGGACCTCTACGCCAACAC	60	0.95
	R-CTGGCTGATCCACATCTGCT		
ssc-miR-451	F-AAACCGTTACCATTACTGAGTT	60	0.92
	R-Uni-miR qPCR Primer, included in kit (miRNA Universal Downstream Primer, TaKaRa)		
U6	F-Uni-miR qPCR Primer, included in kit (TaKaRa)	60	0.93
	R-Uni-miR qPCR Primer, included in kit (TaKaRa)		

TM: melting temperature.

**Table 2:** Correlation analysis of ssc-miR-451 and ACACA expression with pork quality traits.

gene Name	L	a	b	Shear Force	Drip Loss	Cooking Loss	Marbling Score	Crude Protein	IMF ^1^
miR-451	0.304	−0.122	0.522 *	−0.033	0.123	−0.518 *	−0.311	−0.466	−0.551 *
ACACA	−0.193	0.411	−0.565	−0.102	0.001	0.105	0.638 *	0.409	0.706 *

Note: The data of correlation analysis were from 18 DLY pigs. * *p* < 0.05, without * means the difference is not significant. ^1^: IMF: Intramuscular fat content.
